# Alcoholism as a Cause of Crime and Epilepsy

**Published:** 1881-09

**Authors:** 


### ALCOHOLISM AS A CAUSE OF CRIME AND EPILEPSY.

In a recent number of *Brain*, Dr. Clarke has published some statistics, which lead him to the conclusion that "alcoholism of parents is a predisposing cause of crime and epilepsy in their children." Forty-four per cent. of the epileptic criminals were the children of drunken parents. The proportion of epileptic and insane relatives is found to be very much greater with criminals than with ordinary epileptics. The convictions for bastardy are three times as numerous among epileptics as among non-epileptics. The statistics show that the amount of crime, as indicated by the number of convictions, is greater among epileptics than among ordinary criminals.

